# Effect of a Smartphone-Based App on the Quality of Life of Patients With Heart Failure: Randomized Controlled Trial

**DOI:** 10.2196/20747

**Published:** 2020-11-02

**Authors:** Mahboube Davoudi, Tahereh Najafi Ghezeljeh, Farveh Vakilian Aghouee

**Affiliations:** 1 School of Nursing and Midwifery Iran University of Medical Sciences Tehran Iran; 2 Nursing Care Research Center Iran University of Medical Sciences Tehran Iran; 3 Cardiovascular Department Mashhad University of Medical Sciences Mashhad Iran

**Keywords:** heart failure, mobile app, quality of life, mobile phone

## Abstract

**Background:**

Patients with heart failure have low quality of life because of physical impairments and advanced clinical symptoms. One of the main goals of caring for patients with heart failure is to improve their quality of life.

**Objective:**

The aim of this study was to investigate the effect of the use of a smartphone-based app on the quality of life of patients with heart failure.

**Methods:**

This randomized controlled clinical trial with a control group was conducted from June to October 2018 in an urban hospital. In this study, 120 patients with heart failure hospitalized in cardiac care units were randomly allocated to control and intervention groups. Besides routine care, patients in the intervention group received a smartphone-based app and used it every day for 3 months. Both the groups completed the Minnesota Living with Heart Failure Questionnaire before entering the study and at 3 months after entering the study. Data were analyzed using the SPSS software V.16.

**Results:**

The groups showed statistically significant differences in the mean scores of quality of life and its dimensions after the intervention, thereby indicating a better quality of life in the intervention group (*P*<.001). The effect size of the intervention on the quality of life was 1.85 (95% CI 1.41-2.3). Moreover, the groups showed statistically significant differences in the changes in the quality of life scores and its dimensions (*P*<.001).

**Conclusions:**

Use of a smartphone-based app can improve the quality of life in patients with heart failure. The results of our study recommend that digital apps be used for improving the management of patients with heart failure.

**Trial Registration:**

Iranian Registry of Clinical Trials IRCT2017061934647N1; https://www.irct.ir/trial/26434

## Introduction

Heart failure (HF) is a complex and progressive clinical syndrome that causes functional or structural impairments in the heart and results in impaired ventricular emptying or filling [1]. HF was reported in 6.2 million people in the United States of America between 2013 and 2016, which indicates the continuous increase in its prevalence [2]. According to the latest data, the prevalence of HF is expected to increase by more than 46% from 2012 to 2030 [3] owing to increased age and survival rate after a myocardial infarction and increased incidence of risk factors such as diabetes [4].

Patients with HF may experience a wide range of symptoms, including shortness of breath, cough, swelling of extremities, and fatigue [1], which can reduce their physical and mental health capabilities and lead to hospitalization. The rate of hospitalization of these patients is high [5]. Several factors play key roles in the readmission of patients with HF because of illness progression or exacerbated symptoms, including inappropriate treatment adherence and suboptimal health care education during and after the hospitalization period, which results in the lack of knowledge [6]. Patients with HF have low quality of life (QoL) due to their physical problems, advanced clinical symptoms, and readmissions [7]. QoL can be considered as an important indicator of health care, and its improvement is the goal of care in patients with HF [8] for their overall survival. QoL is affected by the health status of patients and is a reflection of a person’s psychological and physical well-being [9]. Poor QoL can be caused by patients’ high rate of hospitalization, morbidity, and mortality. Poor QoL indicates the diverse effects of a health condition and its treatment and caring process on patients’ lives. Thus, improving the QoL of patients with chronic illnesses such as HF is vitally imperative [10].

To improve patients’ QoL, lifestyle changes should be made. In patients with HF, self-care and treatment adherence are the important parts of disease management [4]. One of the reasons for the deterioration of the signs and symptoms in patients with HF is nonadherence, which is related to the lack of knowledge about the illness and treatment process, forgetfulness, complexity of treatment, and personal and social issues [11], as well as a reduction in their motivation to adhere to the therapeutic regimen [12]. Motivating patients and providing quality care by the health care team can increase patients’ adherence to treatment and lead to lifestyle changes [13].

Adequate self-care behavior is shown to result in reduced risk of hospitalizations and mortality among patients with HF [14]. Adherence to self-care behaviors decrease markedly after discharge, which has negative effects on readmission and QoL. Patient education plays a substantial and important role in increasing treatment adherence [15]. Therefore, patients with HF should be educated and supported [14], particularly in the long term after discharge, since they mostly complain of receiving little education and support from health care providers about how to manage their condition, treatment, and self-care [11]. Distance monitoring after discharge from the hospital can help with controlling symptoms, improving self-care [16], and subsequently QoL.

Given the increased prevalence of HF and self-care challenges, the use of simple and accessible technologies to support self-care is important [17]. The long-term nature of the management of chronic diseases and the need for frequent monitoring have motivated researchers to develop advanced therapies and telemonitoring [18]. Recent advances in information technology can provide the potential to remove the barriers to education and clinical monitoring of patients. Since the majority of the worldwide population owns a mobile device, there is inevitable potential for mobile health to provide a platform to access health information and advice [19]. Nowadays, the use of mobile devices has been expanded to provide patients with health care services and purposeful communication between the therapeutic team and patients. The use of telemonitoring by using mobile devices is especially needed to quickly assess the signs and symptoms of the patient and make a decision on the treatment [20].

Use of apps or software on the smartphone can facilitate monitoring of patients’ health through educational messages, audio files, and video clips. Smartphone-based apps have the potential to collect real-time data and graphically draw data for further interactions [21]. In general, although some apps have been developed for patient care, only few studies have examined their effectiveness. The effectiveness of smartphone-based apps for the management of some diseases and conditions have already been evaluated, such as in patients with chronic pain [22], for the diagnosis and notification of acute pesticide poisonings [23], and in patients undergoing heart valve replacement [24]. The aim of this study was to validate a smartphone app called “My Smart Heart” [25] and to investigate its effect on the QoL of patients with HF.

## Methods

### Design of the Trial

This study was a randomized clinical trial (pretest and posttest with a control group design, Multimedia Appendix 1), which was conducted in 2018 in the cardiac care units of Mashhad Medical Center, Iran. After obtaining approval from the ethics committee affiliated with the Iran University of Medical Sciences (code: 1396.9411449003) and registration on the website of clinical trials (code: IRCT2017061934647N1), willing patients signed the letter of consent before the study. This study adhered to the basic ethics principles and the tenets of the Declaration of Helsinki.

### Patient Recruitment

In this study, patients with HF who were admitted to the cardiac care unit were selected using the following inclusion criteria: age of 18-65 years, being literate, class II or III of HF according to the New York Heart Association classification, patients admitted to the hospital due to exacerbation of HF, having a smartphone or a tablet with the Android operating system, and ability to use a smartphone and the app. Patients who met the following criteria were excluded from analysis: unwillingness to continue participation in the study, no use of the app for 1 week (each patient used a one-time password to enter the software), and death.

**Figure 1:**
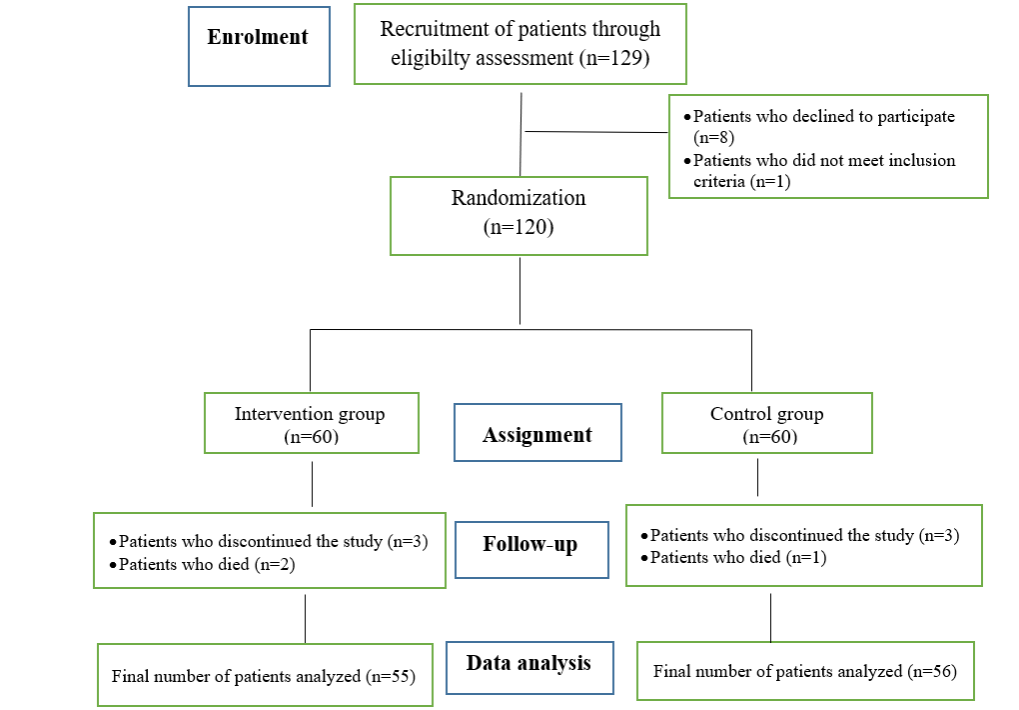
The process of this study.

The sequential sampling method was used. The randomized block method with the ratio of 1:1 and no permutation was used to assign the patients to the groups. Different modes of assignments to the groups were written on 4 cards and placed in opaque envelopes. Next, the envelopes were placed in a box. A research collaborator who was unaware of the assignment process took the envelopes from the box and determined each patient’s place in the groups. This process was continued until the desired sample size was achieved. It should be noted that due to the nature of the intervention, there was no possibility of blinding the subjects. To estimate the sample size at 95% confidence interval, 80% power, assuming that the effect size of the intervention on the QoL in the intervention group compared to the control group would be at least *d*=10.5 and SD=18 [26] and with 25% possibility of sample dropout, the sample size was considered as 60 people in each group (Figure 1).

### Data Collection

Data were collected from the patients’ medical records and through interviews using the demographic data form, health information form, and Minnesota Living with Heart Failure Questionnaire (MLHFQ).

Demographic data form: A researcher-made demographic form was prepared on the basis of literature review. It was completed before the intervention by examining a patient’s medical file and interviewing him/her. The content validity of this form was assessed and approved by an expert panel.

Health information form: This was a researcher-designed form that was prepared on the basis of literature review. This form was completed before the intervention by examining the patient's medical file and interviewing him/her. The content validity of this form was assessed and approved by an expert panel.

MLHFQ: The MLHFQ is the most common tool for investigating the QoL of patients with HF [27]. The validity and reliability of the Farsi version of this tool have been assessed and approved using the calculation of the Cronbach α coefficient of .95 and reliability score of 0.90 [28]. This questionnaire assesses the patients’ perceptions of the effects of dysfunctional illness on their physical, economic, social, and psychological functions. This questionnaire consisted of 21 items on the 6-point Likert scale from 0 (some limitations) to 5 (maximum limitations). The score range was between 0 and 105, with a high score indicating a poor QoL. In this questionnaire, 9 questions were related to physical function, 5 questions covered the psychoemotional aspect, and 7 questions examined the socioeconomic conditions. The questionnaire was designed to be opened and filled out using the Google platform on their smartphones. The patients were asked to fill the questionnaire, and those with a poor and moderate QoL and a score more than 24 entered the study. Further, 3 months after the starting point of the study, the same questionnaire was sent to the patients via the smartphone’s virtual social network to be filled out. A follow-up phone call was made to ask the patients to fill the questionnaire. Finally, for reliability, the Cronbach α coefficient was calculated to be .71. The patients were asked to fill the questionnaires, and a researcher who was blinded to the study process gathered these questionnaires at the beginning of this study and at 3 months after the patients entered the study.

### Smartphone App Features

**Figure 2:**
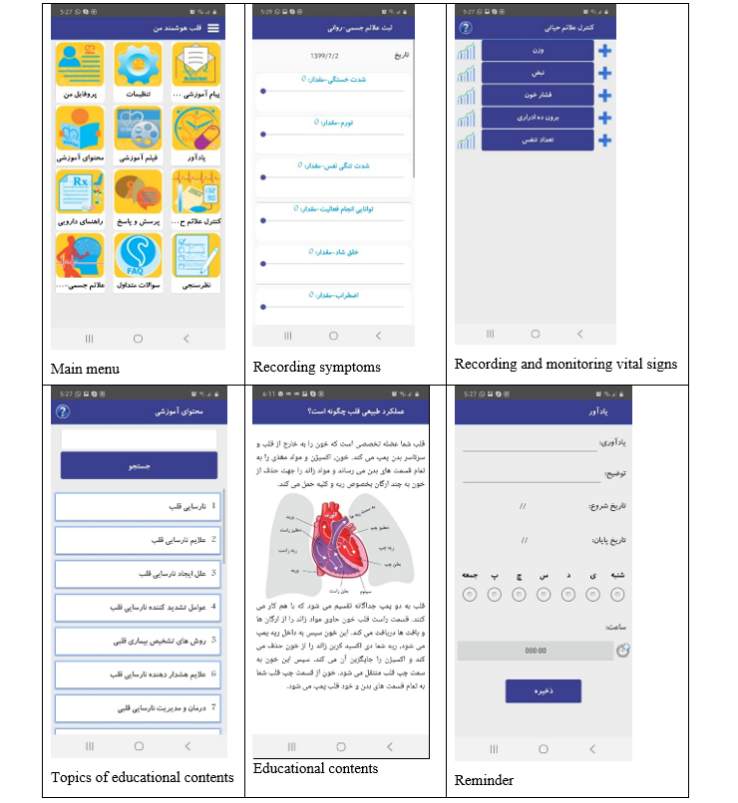
Features of the interactive Android-based smartphone app.

An Android-based smartphone app was developed and evaluated [25]. This interactive app (Figure 2) can be used both online and offline. The main characteristics of this app are profiles, reminders, educational content, educational videos, daily messages, pharmacy guides, frequently asked questions, daily recording of physical and psychological symptoms, and vital signs and sending alerts as needed. This app was evaluated by patients, health care providers, nurses, physicians, and programmers, thereby indicating its simple and convenient use and consistency with the principles of the American Heart Failure Association and the Institute of Health Information Technology [25]. This app has the following features: (1) informing: information about disease, symptoms, symptom management, treatment, and care in various formats (text, photos, and videos); (2) instruction: provision of instructions for the user of the app; (3) recording: ability to record user’s information under the subsets of collecting information, sharing information with the researcher, evaluating information, and intervening, as needed; (4) displaying: displaying data regarding symptoms, vital signs, and weights graphically and textually, as well as the ability to extract the PDF and XML formats; (5) guiding: provision of guidance based on the data given by the user and provision of appropriate advice through communication with the researcher; (6) reminder or alert: reminding the user regarding the time of taking medicines, performing tests, and other requirements; and (7) interaction: bilateral interaction between the researcher and patient [25]. This app was provided to patients to use for 3 months.

### Intervention

Patients in the intervention group received the smartphone-based app besides routine care. It was installed on their Android phones, and the patients were taught how to use the program via a 30-minute face-to-face session. The app brochure was also provided to the patients. The method of measuring vital signs, monitoring symptoms, and recording them in the app were taught to patients via the educational content of the app. Every week for 6 consecutive weeks and then every month for about 2 months, notifications were sent to the patients to remind them to use the app. Patients were asked to enter their daily vital signs, symptoms, and weight in the app, which provided the opportunity for health care providers to telemonitor the changes in the data in the text and graph format. In addition, patients could evaluate their daily conditions and its changes by visualizing the recorded data in the shape of a graph. Further, the daily use of the app by the patients was evaluated by the management panel on the internet and encouragements for the use of the app were provided, as needed. Patients and researchers could interact with each other depending on the patients’ needs. During the app usage period (3 months), the patients were supported in terms of how to use the program. To provide support, the researcher’s telephone number was provided to the patients for contact purposes, if needed. The patients in the control group received routine care, which included the provision of a brochure and the method of taking medicines and referral to the doctor at a clinic 2 weeks later. Moreover, the patients in the control group were provided with the smartphone-based app after data collection.

### Data Analysis

Descriptive statistics (frequency, mean, and SD) were used to analyze the qualitative and quantitative data. The Kolmogorov-Smirnov test was used to assess the normal distribution of the quantitative variables. The two-sided *t* test was used to compare the quantitative variables. The chi-square and Fisher exact tests were used for comparing the qualitative variables between the 2 groups. The two-factor analysis of variance was used for determining the effect of the demographic and health information on the QoL in order to investigate the effects of 2 factors (intervention and demographic and health information) on the dependent variable. The repeated measure analysis of variance test was used to determine the effect of the intervention over time. The Cohen effect was used to assess the effect of the intervention. All statistical tests were performed using the SPSS V.22 software (IBM Corp). The significance level was set as *P*<.05.

## Results

In this study, data collected from 111 patients were used for the analysis. The groups showed no statistically significant differences before the study regarding demographic characteristics (Table 1).

Table 2 shows that before the study, the mean score of QoL in the intervention and control groups were 42.91 (15.62) and 47.42 (16.38), respectively. The independent two-sided *t* test showed that the groups had no statistically significant differences before the study in any of the dimensions (*P*=.14). At 3 months after the intervention (Table 2), the mean scores of QoL in the intervention and control groups were 26.03 (9.67) and 50.13 (15.54), respectively. The groups had statistically significant differences in the QoL after the intervention, with a better QoL in the intervention group (*P*<.001). According to the results, the effect size of the intervention was high. The mean scores of QoL after the intervention in the intervention group in all domains were less than those in the control group, indicating better QoL in the intervention group compared to that in the control group (*P*<.001). The effect size of the intervention was high. In the intervention group, the reduced scores indicated improved QoL in all the domains compared to that in the control group. In the intervention group, the total and all the domain mean scores of QoL before and after the intervention had a statistically significant difference, indicating an improved QoL after the use of the app (*P*<.001). In the control group, the total mean score of QoL before and after the intervention showed a statistically significant difference (*P*=.001). The mean score of QoL in the control group in all dimensions before and after the intervention showed a statistically significant difference, indicating a reduced QoL in all domains over 3 months.

**Table 1:** Demographic and health information of the patients with heart failure before the intervention.

Variables, subgroups		Intervention group, n=55	Control group, n=56	*P* value	
**Sex, n (%)**					.38
	Male	34 (62)	30 (54)		
	Female	21 (38)	26 (46)		
**Age (years)**					.24
	<40, n (%)	10 (18)	7 (13		
	40-49, n (%)	14 (25)	12 (22)		
	50-59, n (%)	15 (27)	12 (21)		
	>60, n (%)	16 (29)	25 (45)		
	Mean (SD)	50.07 (11.77)	52.78 (12.2)		
**Education level, n (%)**					.18
	Elementary to high school	25 (46)	35 (63)		
	After high school and before entering university	19 (35)	15 (27)		
	University degree	11 (20)	6 (10)		
**Marital status, n (%)**					.18
	Married	51 (93)	46 (82)		
	Single	4 (7)	10 (18)		
**Employment status, n (%)**					.69
	Unemployed	4 (7)	7 (13)		
	Housewife	20 (36)	24 (43)		
	Employed	15 (27)	13 (23)		
	Retired	4 (7)	7 (13)		
**Income adequacy, n (%)**					.31
	Adequate	19 (35)	13 (23)		
	Somewhat adequate	20 (36)	20 (36)		
	Not adequate	16 (29)	23 (41)		
Time since diagnosis (years), mean (SD)		4.52 (4.41)	4.57 (5.01)	.96	
Body mass index, mean (SD)		25.96 (3.83)	25.42 (4.59)	.50	
**Cigarette smoking, n (%)**					.97
	Yes	8 (15)	8 (14)		
	No	47 (85)	48 (86)		
**Comorbidities, n (%)**					.36
	No	17 (31)	13 (23)		
	Yes	38 (69)	43 (77)		
**Types of comorbidities, n (%)**					
	Diabetes	19 (50)	26 (61)	.34	
	Hypertension	28 (74)	33 (77)	.75	
	Pulmonary disease	5 (13)	5 (13)	.71	
**Previous hospitalization in the last 3 months because of heart failure, n (%)**					
	Yes	31 (56)	33 (59)	.12	
	No	24 (44)	23 (41)	.11	
**Frequency of previous hospitalization in the last 3 months, n (%)**					.08
	1 time	18 (55)	10 (42)		
	2 times	11 (33)	5 (21)		
	3 times or more	4 (12)	9 (38)		
Ejection fraction, mean (SD)		29.41 (9)	29.19 (9)	.90	

**Table 2:** Comparison of the mean scores of the quality of life before and after the intervention between and within the groups.

Parameters, Domain/times			Intervention group, n=55		Control group, n=56		Independent *t* *(df)* test		*P* value
**Physical function**									
	Before, mean (SD)	18.87 (6.73)		21.75 (8.76)		1.937 (109)		.055	
	After, mean (SD)	11.96 (5.44)		22.71 (8.05)		8.226 (109)		<.001	
	Paired *t* *(df)* test	7.23 (54)		2.274 (55)		N/A^a^		N/A	
	*P* value	<.001		.03		N/A		N/A	
	Changes, mean (SD)	–6.90 (7.08)		0.96 (3.17)		7.53 (74.52)		<.001	
**Psychological function**									
	Before, mean (SD)	9.26 (5.41)		10.96 (5.10)		1.703 (109)		.09	
	After, mean (SD)	4.14 (2.77)		11.71 (4.80)		10.149 (109)		<.001	
	Paired *t* *(df)* test	7.26 (54)		2.803 (55)		N/A		N/A	
	*P* value	<.001		.007		N/A		N/A	
	Changes, mean (SD)	–5.11 (5.22)		0.75 (2.00)		7.836 (69.27)		<.001	
**Social function**									
	Before, mean (SD)	14.76 (5.77)		14.68 (6.29)		0.072 (109)		.94	
	After, mean (SD)	9.92 (4.21)		15.7 (5.60)		6.127 (109)		<.001	
	Paired *t* *(df)* test	5.59 (54)		2.667 (55)		N/A		N/A	
	*P* value	<.001		.01		N/A		N/A	
	Changes, mean (SD)	–4.83 (6.41)		1.02 (2.86)		6.228 (74.41)		<.001	
**Total**									
	Before, mean (SD)	42.91 (15.62)		47.42 (16.38)		1.483 (109)		.14	
	After, mean (SD)	26.03 (9.67)		50.13 (15.54)		9.787 (109)		<.001	
	Paired *t* *(df)* test	7.82 (54)		3.55 (55)		N/A		N/A	
	*P* value	<.001		.001		N/A		N/A	
	Changes, mean (SD)	–16.88 (16.00)		2.71 (5.71)		8.617 (67.34)		<.001	

^a^N/A: not applicable.

Table 3 shows that there was significant correlation between the QoL and income adequacy and previous hospitalization in the last 3 months among the patients. Two-factor analysis of variance indicated that these 2 independent variables had no significant effect on the QoL (Table 4).

**Table 3:** Correlation of the mean scores of the quality of life with the demographic and health information of patients with heart failure.

Variables, groups		Quality of life		
		Mean (SD)	*P* value	
**Sex**				.51^a^
	Male	46.06 (17.56)		
	Female	44.00 (13.95)		
Age (years)		N/A^b^	.74^c^	
**Education level**				.76^d^
	Elementary to high school	46.20 (15.69)		
	After high school and before entering university	43.64 (16.06)		
	University degree	44.7 (18.23)		
**Marital status**				.08^a^
	Married	52.35 (12.7)		
	Single	44.15 (16.32)		
**Employment status**				.48^d^
	Unemployed	50.81 (16.29)		
	Housewife	42.75 (12.9)		
	Employed	45.71 (19.09)		
	Retired	46.29 (17.42)		
**Income adequacy**				.02^d^
	Adequate	40.29 (14.92)		
	Somewhat adequate	43.57 (14.72)		
	Not adequate	50.86 (17.02)		
Time since the diagnosis (years)		N/A	.08^c^	
Body mass index		N/A	.64^c^	
**Cigarette smoking**				.20^a^
	Yes	44.38 (15.83)		
	No	49.98 (17.32)		
**Comorbidities**				.89^a^
	No	45.53 (17.23)		
	Yes	45.06 (15.77)		
**Previous hospitalization in last 3 months because of heart failure**				<.001^a^
	Yes	38.26 (10.45)		
	No	52.50 (17.78)		
Ejection fraction		N/A	.07^c^	

^a^Independent *t* test.

^b^N/A: not applicable.

^c^Pearson correlation test.

^d^Analysis of variance test.

**Table 4:** Results of the two-factor analysis of variance.

Variables, Domain/times		Intervention group, n=55, mean (SD)	Control group, n=56, mean (SD)	Partial eta squared		
				Group variable	Income	Hospitalization
**Physical function**						
	Before	18.87 (6.73)	21.75 (8.76)	0.066 (0.034)	0.185 (0.034)	0.169 (0.025)
	After	11.96 (5.44)	22.71 (8.05)	0.000 (0.357)	0.343 (0.021)	0.396 (0.007)
**Psychological function**						
	Before	9.26 (5.41)	10.96 (5.10)	0.098 (0.027)	0.213 (0.031)	0.105 (0.026)
	After	4.14 (2.77)	11.71 (4.80)	0.000 (0.473)	0.088 (0.048)	0.399 (0.007)
**Social function**						
	Before	14.76 (5.77)	14.68 (6.29)	0.999 (0.000)	0.112 (0.043)	0.315 (0.010)
	After	9.92 (4.21)	15.7 (5.60)	0.000 (0.235)	0.134 (0.040)	0.614 (0.003)
**Total**						
	Before	42.91 (15.62)	47.42 (16.38)	0.146 (0.021)	0.087 (0.048)	0.111 (0.025)
	After	26.03 (9.67)	50.13 (15.54)	0.000 (0.449)	0.107 (0.044)	0.366 (0.008)

## Discussion

### Principal Findings

In this study, according to the findings, changes in the QoL score and its dimensions in the intervention group were more than those of the control group, and the effect size of the intervention was high. Therefore, use of a smartphone-based app increased the QoL and its dimensions in patients with HF. It is believed that new technologies such as apps can increase patients’ satisfaction with medical care and improve their relationships with health care staff and subsequently improve their QoL [29]. Such programs can increase the motivation of the patients to make changes in their lifestyles and increase their QoL. Further, any intervention that reduces the rate of hospitalization and symptoms in patients with HF can improve their QoL. A smartphone app that can record symptoms and provide intelligent responses to the patients’ symptoms and related changes in each week and month can help them manage their symptoms effectively. This app can cause a significant improvement in patients’ QoL [29]. The app provides the possibility of continuous monitoring of patients even if they are not hospitalized. Moreover, this app and its use are not limited to a specific time and place [30]. In this study, there was a possibility to contact the health care provider via this app, and caregivers could help with reducing the patient’s or family’s ambiguities with regard to the patient’s health condition.

Interventions that improve QoL are integral parts of the management of HF [9]. They can be performed by the therapist as a continuous process of follow-up and monitoring. However, in a review study, wherein the effect of the use of mobile-based apps on the QoL of patients with HF was studied, only a limited number of apps had the required criteria for caring of patients with HF and most programs were commercial rather than functional. Moreover, app use had little impact on the QoL of patients [31]. In the production of the “My Smart Heart” app, efforts were made to reduce the limitations of the existing apps and improve the QoL of patients. The results of our study shows that the effect of this intervention on the QoL of patients with HF was high. In line with the results of this study, a study after 6 months of intervention in patients with myocardial infarction found that the mean score of QoL in the intervention group increased compared to that in the control group [32].

### Strengths and Limitations

The smartphone-based app used in our study provides a novel telemonitoring and education method for patients with HF. This study was conducted on those patients who were able to communicate and were literate, with an ejection fraction of less than 45%. For generalizability, similar studies should be conducted in illiterate patients. In this study, the effect of the intervention was studied after 3 months, but the long-term effects of the intervention need to be further researched. In this study, the mediator effects of knowledge and self-care improvement were not evaluated, and therefore, these should be investigated in future studies. Within 3 months of the study process, some medication errors were identified. Therefore, studies should be performed to demonstrate the efficacy of software in improving patient safety and reducing medication errors.

### Conclusions

Our study showed that the use of a smartphone-based app increased the QoL of patients with HF. Interventions that improve QoL constitute an integral part of the management of HF, which can be carried out by the health care provider through continuous follow-up of patients using such apps. Nurses as well as patients and their families can use smartphone-based interactive software. Provision of facilities such as counselling centers can provide patients and their families with information about how to use this software to improve their QoL. The results of this research can be considered by nursing managers for patient education. However, it is necessary to study the cost-effectiveness of such interactive software. This method can be used by nurses and other health care providers in HF clinics and heart transplantation centers to improve patients’ and families’ satisfaction with health care, which needs to be further studied in the future.

## Appendix

Multimedia Appendix 1 CONSORT-eHEALTH checklist (V1.6.1).

## Abbreviations

HF: heart failure
MLHFQ: Minnesota Living with Heart Failure Questionnaire
QoL: quality of life
